# Polyhydroxyalkanoate Biosynthesis at the Edge of Water Activitiy-Haloarchaea as Biopolyester Factories

**DOI:** 10.3390/bioengineering6020034

**Published:** 2019-04-16

**Authors:** Martin Koller

**Affiliations:** 1 Office of Research Management and Service, c/o Institute of Chemistry, University of Graz, NAWI Graz, Heinrichstrasse 28/III, 8010 Graz, Austria; martin.koller@uni-graz.at; Tel.: +43-316-380-5463; 2 ARENA—Association for Resource Efficient and Sustainable Technologies, Inffeldgasse 21b, 8010 Graz, Austria

**Keywords:** Archaea, bioeconomy, biopolyester, downstream processing, extremophiles, haloarchaea, *Haloferax*, halophiles, polyhydroxyalkanoates, salinity

## Abstract

Haloarchaea, the extremely halophilic branch of the Archaea domain, encompass a steadily increasing number of genera and associated species which accumulate polyhydroxyalkanoate biopolyesters in their cytoplasm. Such ancient organisms, which thrive in highly challenging, often hostile habitats characterized by salinities between 100 and 300 g/L NaCl, have the potential to outperform established polyhydroxyalkanoate production strains. As detailed in the review, this optimization presents due to multifarious reasons, including: cultivation setups at extreme salinities can be performed at minimized sterility precautions by excluding the growth of microbial contaminants; the high inner-osmotic pressure in haloarchaea cells facilitates the recovery of intracellular biopolyester granules by cell disintegration in hypo-osmotic media; many haloarchaea utilize carbon-rich waste streams as main substrates for growth and polyhydroxyalkanoate biosynthesis, which allows coupling polyhydroxyalkanoate production with bio-economic waste management; finally, in many cases, haloarchaea are reported to produce copolyesters from structurally unrelated inexpensive substrates, and polyhydroxyalkanoate biosynthesis often occurs in parallel to the production of additional marketable bio-products like pigments or polysaccharides. This review summarizes the current knowledge about polyhydroxyalkanoate production by diverse haloarchaea; this covers the detection of new haloarchaea producing polyhydroxyalkanoates, understanding the genetic and enzymatic particularities of such organisms, kinetic aspects, material characterization, upscaling and techno-economic and life cycle assessment.

## 1. Introduction

The first description of a biological polymer with plastic-like properties was published in the 1920s, when Maurice Lemoigne detected light-refractive intracellular inclusion bodies [1], today referred to as “granules”—or, more recently “carbonosomes” [2]—n resting cultures of the Gram-positive bacterium *Bacillus megaterium*. Based on the acidic degradation product of these inclusions, 3-hydroxybutyrate (3HB), Lemoigne correctly assumed the microscopically observed intracellular product to be the polymer of 3HB, namely poly(3-hydroxybutyrate) (PHB). In the meantime, PHB and its related homo- and heteropolyesters, as a group labelled as polyhydroxyalkanoates (PHA), have attracted global attention as biological, bio-based, biocompatible and biodegradable alternatives to established plastics of petrochemical origin in many sectors of the rocketing plastic market [3,4]. PHA consist of a variety of diverse building blocks, which make their material properties highly versatile [5], and can be produced biotechnologically by different continuous or discontinuous fermentation approaches and feeding strategies [6]. In principle, short chain length PHA (*scl*-PHA) are distinguished from medium chain length PHA (*mcl*-PHA). While *scl*-PHA typically constitute thermoplastic materials, *mcl*-PHA are known as materials with elastomeric and latex-like properties and are often of a sticky nature. Among *scl*-PHA, the homopolyester PHB and the copolyester poly(3-hydroxybutyrate-*co*-3-hydroxyvalerate) (PHBHV) are best described; in this context, increasing 3-hydroxyvalerate (3HV) fractions in the copolyester decreases melting temperature and crystallinity, which makes such PHBHV copolyesters easier to process than the rather crystalline and brittle PHB, a material of restricted applicability [3,4].

**Figure 1:**
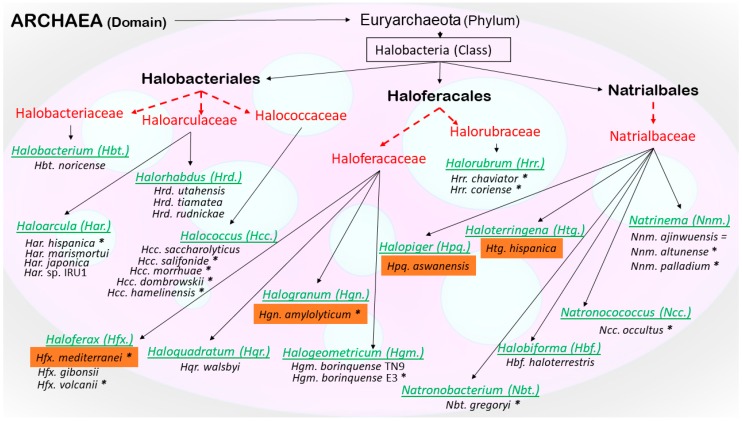
Extract from the phylogenetic tree of haloarchaea, selecting those species reported to accumulate PHA biopolyesters. Colored (orange) background highlights the limited number of species to date cultivated on bioreactor scale to study PHA production. The asterisks indicate 3-hydroxyvalerate production by the strain from structurally unrelated substrates. (Bold: orders; red: families; *italics and underlined*: genera; *italics*: species).

Apart from wild-type and genetically engineered eubacteria and recombinant yeasts, plants, and microalgae, PHA biosynthesis takes place also in the cytoplasm of various extremely halophilic species from the Archaea domain, the so called “haloarchaea”. Exclusively *scl*-PHA production is reported for haloarchaea, while for eubacteria, both *scl*- and *mcl*-PHA production is reported [3,4]. Extremely challenging habitats include environments where such highly adaptive survivalists are typically isolated; illustrative examples are the Great Salt Lake, the Dead Sea, hypersaline anoxic deep-sea basins, solar saltern crystallizers, hypersaline soil samples, salt mine boreholes, salt production pans, or even alpine dry salt rocks. The taxonomic classification of these extremely salt-demanding, typically aerobic organisms is by no means a trivial task and is based on steadily refined knowledge about the genomics, proteomics, metabolomics, and lipidomics of these organisms. Traditionally, haloarchaea are members of the family Halobacteriaceae, which belongs to the order Halobacteriales, which in turn is part of class III (*Halobacteria*) consisting of two major clades A and B, of the phylum and (sub)kingdom of Euryarchaeota, which belongs to the domain of Archaea (according to International Committee on Systematics of Prokaryotes, Subcommittee on the taxonomy of Halobacteriaceae; cited by [7]). Later, members of the class *Halobacteria* were re-grouped into three orders: a revised order Halobacteriales and two new orders, Haloferacales and Natrialbales, which encompass the novel families Haloferacaceae and Natrialbaceae [8]. More recently, based on phylogenetic analyses and conserved molecular characteristics, it was suggested to divide the order “Halobacteriales” into the families Halobacteriaceae, Haloarculaceae, and Halococcaceae, and the order “Haloferacales” into the families Haloferacaceae and Halorubraceae [9]. These are the currently valid designations of the families where haloarchaea demonstrated to produce PHA are grouped. Figure 1 provides a schematic overview about the phylogenetic classification of the haloarchaeal species discussed in the present review.

Talking about PHA biosynthesis by haloarchaea, it took the scientific community until 1972, when Kirk and Ginzburg carried out morphological characterizations of a Dead Sea isolate, which was labeled “*Halomonas* sp.” by these authors. This organism was cultivated on a highly saline medium containing 200 g/L NaCl. By using freeze-fracture and freeze-etch techniques, the authors revealed plastic-like cytoplasmic inclusion bodies, which were extracted from microbial biomass and investigated by X-ray diffractometry. Grounded solely on these examinations, the authors correctly recognized this material as the biopolyester PHB, the material already known at the time as a carbon and energy storage product for many eubacteria, as reported by Lemoigne [1] and succeeding generations of scholars. In any case, this study by Kirk and Ginzburg was the very first unambiguous description of PHA production by an archaeon [10]. Regarding the production strain “*Halomonas* sp.”, it took nearly three decades until this isolate was classified as *Haloarcula* (*Har.*) *marismortui*, its currently valid species name, in a report published by Nicolaus et al. [11].

## 2. Genetic and Enzymatic Particularities of Haloarchaeal PHA Biosynthesis

Generally, PHA synthases, the enzymes catalyzing the polymerization of PHA precursors (hydroxyacyl-CoAs like acetyl-CoA, propionyl-CoA, etc.) found in haloarchaea are grouped in the Class III of PHA synthases [12]. Class III PHA synthases were identified in several eubacteria such as *Allochromatium vinosum* (previously known as *Chromatium vinosum*) or *Thiocapsa pfennigii*; Class III PHA synthases polymerize short hydroxyacyl-CoAs, namely those not longer than 3-hydroxyvaleryl-CoA; moreover, such synthases are typically composed of two subunits: the catalytically active subunit PhaC (molar mass ranging from 40–53 kDa) and the structural subunit PhaE (molar mass 20–40 kDa), which is also indispensable for polymerization. Together, the two subunits form a biocatalytic cluster, the so called “PhaEC complex” [13]. Hezayen et al. were the first scientists who revealed the special features of PHA synthases in haloarchaea. When studying “strain 56” (today classified as *Halopiger* (*Hpq.*) *aswanensis*), a species isolated from hypersaline soil collected near Aswan, Egypt, which thrives best with 250 g/L NaCl, Hezayen et al. discovered a PHA synthase covalently bound to the PHA granules. This enzyme exposed particular features in comparison to PHA synthases in eubacteria described earlier; the new enzyme displayed high thermostability up to 60 °C, with strongly increasing activity at higher salinity; especially, Mg^2+^ ion concentration had a significant effect on synthase activity. In addition, this halophilic biocatalyst exhibited a remarkably narrow spectrum of PHA-precursors: the enzyme polymerized only 3-hydroxybutyryl-CoA, but neither 3-hydroxyvaleryl-CoA, 4-hydroxybutyryl-CoA, nor 3-hydroxyhexanoyl-CoA. Most extraordinarily, no other PHB biosynthesis enzymes typically needed for PHA biosynthesis in bacterial PHA production strains, namely 3-ketothiolase or NADH/NADPH-dependent acetoacetyl–CoA reductase, were produced by *Hpq. aswanensis*; this evidenced for the first time that haloarchaea use a metabolic route for PHA biosynthesis different to eubacteria [14].

To better comprehend and to enhance PHA production by haloarchaea, various subsequent genomic and enzymatic investigations were carried out. Similar to the studies with *Hpq. aswanensis*, the haloarchaeal genes encoding for homologues of bacterial Class III PhaC synthase enzymes were identified by Baliga et al. also in the genomes of *Har. marismortui* isolated from the Dead Sea [15], or by Bolhuis et al. in “Walsby’s square bacterium” *Haloquadratum* (*Hqr.*) *walsbyi* isolated from different saltern crystallizers [16]. Han and colleagues, active at Professor Xiang´s laboratories, which are world-leading in the study of PHA biosynthesis genes in haloarchaea, explored for the first time the expression profile of genes encoding haloarchaeal PHA synthases. In this work, *Har. marismortui*, when cultivated in defined saline medium containing high amounts of glucose as carbon source, is able to accumulate PHB fractions in cell dry mass (CDM) up to 21 wt.%. As a major result, the neighboring genes *phaE_Hm_* and *phaC_Hm_* were identified by molecular characterization of the *phaEC_Hm_* operon; these two genes encode two Class III PHA synthase subunits, and are triggered by only one single promoter. It was shown that these genes are constitutively expressed, both under balanced and nutrient-limited cultivation conditions. Remarkably, in contrast to the non-granule associated gene *PhaE_Hm_*, *PhaC_Hm_* is strongly connected to the PHA granules. Inserting *phaE_Hm_* or *phaC_Hm_* genes into the closely related strain *Har. hispanica*, which contains highly homologue *phaEC_Hh_* genes, considerably increased PHB biosynthesis. Particularly, the co-expression of both genes resulted in the highest PHB productivity; in contrast, deleting *phaEC_Hh_* genes from the *Har. hispanica* genome (“knocking out”) totally terminates PHA production. By transferring *phaEC_Hm_* genes into such knockout mutants fully restored the activity of PHA synthase and PHA accumulation. These studies validated for the first time the high significance of *phaEC* genes for PHA biosynthesis in haloarchaea [17].

Lu et al. carried out groundbreaking work with *Haloferax* (*Hfx.*) *mediterranei* to elucidate the genetic and enzymatic PHA biosynthesis background of this strain. Using thermal asymmetric interlaced PCR, these authors were able to clone the *phaEC_Hme_* gene cluster of strain *Hfx. mediterranei* CGMCC 1.2087. By Western blotting, it was shown that, analogous to the above described findings for *Har. marismortui*, both *phaE_Hme_* (about 21 kDa) and *phaC_Hme_* (about 53 kDa) genes were constitutively expressed, and both synthases were strongly connected to the PHA granules. Interestingly, the strain synthesized poly(3-hydroxybutyrate-*co*-3-hydroxyvalerate) (PHBHV) copolyesters in both nutrient-limited (supplemented with 1% starch, production of up to 24 wt.% PHBHV in CDM) and nutrient-rich (up to 18 wt.% PHBHV in CDM) media in shaking flask experiments. Knockout of *phaEC_Hme_* genes in this strain completely stopped PHBHV biosynthesis; PHBHV biosynthesis capability was re-established only after complementation with the complete *phaEC_Hme_* gene cluster, but not when transferring either *phaE_Hme_* or *phaC_Hme_* alone. It is worth noting that the described PhaC synthase subunits were considerably longer at their carbon-end than reported for bacterial PHA synthases; this C-terminal extension of *PhaC_Hme_* was shown to be indispensable for the enzymes´ in vivo activity at high salinity. Moreover, a 1:1 mixture of isolated *PhaE_Hme_*/*PhaC_Hme_* enzymes displayed substantial PHA synthesis activity in vitro. These outcomes showed that also *Hfx. mediterranei* possesses the novel type of class III PHA synthases typical for haloarchaea, which are assembled by *PhaC_Hme_* and *PhaE_Hme_* subunits [18]; this corresponds to the above described discoveries for PHA synthases in *Har. hispanica* and *Har. marismortui* [17].

By further sequencing of the *Hfx. mediterranei* CGMCC 1.2087 genome, Han and colleagues identified three more “hidden” *phaC* genes (*phaC1*, *phaC2*, and *phaC3*), which encode possible PhaC synthases. The three “cryptic” genes were distributed all over the whole *Hfx. mediterranei* genome. Similar to PhaC_Hme_ (molar mass 54.8 kDa), PhaC1 (49.7 kDa) and PhaC3 (62.5 kDa) exhibited conserved Class III PHA synthase motifs, which was not the case for PhaC2 (40.4 kDa). Moreover, the longer C-terminus of the other three PhaC enzymes was not found in PhaC2. It was revealed via reverse transcription PCR (RT-PCR) that among all four genes, only *phaC_Hme_* was transcribed in the wild-type strain under conditions supporting PHA biosynthesis. Astonishingly, heterologous co-expression of *phaE_Hme_* with each *phaC* gene in the PHA-negative mutant *Har. hispanica* PHB-1 revealed that all PhaCs, except PhaC2, effect PHBHV synthesis, though with different 3HV portions in the copolyesters. These products were characterized, revealing that thermal properties (melting point, crystallinity, glass transition temperature, etc.) and molecular mass strongly depend on the 3HV fraction in the copolyester. Briefly, the study defined three novel “hidden” *phaC* genes in *Hfx. mediterranei*, and suggested that genetic engineering of these “cryptic” *phaC* genes might have biotechnological applicability in terms of designing PHBHV copolyesters of tailored material properties based on the fine-tuning 3HV contents [19]. In 2012, Han et al. were able to report the complete genome sequence of *Hfx. mediterranei* CGMCC 1.2087, which has a size of 3,904,707 bp and consists of one chromosome and three mega-plasmids, by using a combination of 454 pyrosequencing and Sanger sequencing [20]. Shortly thereafter, Ding et al. deciphered the complete sequence of the *Har. hispanica* ATCC 43049 genome; unexpectedly, these authors noticed substantial differences when comparing this sequence with the gene sequence of *Har. hispanica* ATCC 33960 [21], the model organism used for molecular characterization studies by Han et al. described above [19]. In any case, the works presented by Han et al. [19] and Ding et al. [21] demonstrate that clustered *phaEC* genes encoding Class III PHA synthases are typical features of PHA-producing haloarchaea. This was substantiated by Han and colleagues, who screened PHA synthase genes in haloarchaeal PHA producers from 12 genera; the authors demonstrated the wide distribution of *phaEC* genes among haloarchaea. Compared to their bacterial counterparts, haloarchaeal PHA synthases differ significantly in both molecular weight and some conserved motifs. Therefore, Han and colleagues proposed to classify haloarchaeal PHA synthases as “subtype IIIA”, while type III PHA synthases from bacteria were proposed as “subtype IIIB” [22].

**Figure 2:**
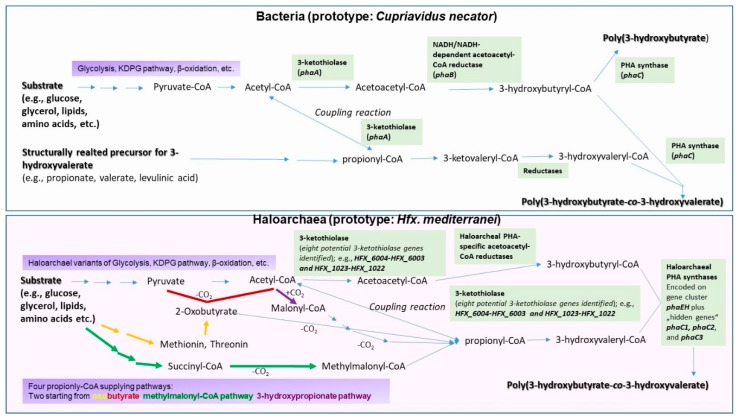
Simplified illustration of PHB and PHBHV biosynthesis by eubacteria (upper part, in grey; prototype organism: *C. necator*) and haloarchaea (lower part, in pink; prototype: *Hfx. mediterranei*). Enzymes and genes (in italics) involved in the PHA biosynthesis steps (starting from acetyl-CoA and propionyl-CoA) are in green text boxes. Special emphasis is dedicated to the propionyl-CoA supplying pathways in haloarchaea: Propionyl-CoA is generated (a) beginning with the coupling of pyruvate and acetyl-CoA, and the decarboxylation of 2-oxobutyrate (marked in brown), (b) starting from the conversion of the amino acids methionine or threonine to 2-oxobutyrate (marked in yellow), (c) starting from succinyl-CoA via methlymalonyl-CoA (marked in green), or (d) starting with carboxylation of acetyl-CoA to malonyl-CoA (marked in purple). Based on [26].

Genome analysis of *Hfx. mediterranei* has also evidenced eight potential 3-ketothiolase genes in *H. mediterranei*, which might express enzymes responsible for the condensation of two acetyl-CoA molecules to acetoacetyl-CoA, or one acetyl-CoA and one propionyl-CoA to 3-ketovaleryl-CoA. It was shown that only the 3-ketothiolases encoded by *HFX_6004-HFX_6003* and *HFX_1023-HFX_1022* are involved in the biosynthesis of PHBHV. Knockout of *HFX_6004-6003* leads to the accumulation of PHB homopolyester without the 3HV building blocks, while simultaneous knockout of *HFX_6004-6003* and *HFX_1023-1022* stopped the strain´s ability to produce PHA. This was the first report on haloarchaeal 3-ketothiolases, which reveled considerable differences to their bacterial relatives in subunit composition and catalytic residue [23]. Finally, genes encoding for PHA-specific acetoacetyl-CoA reductases, catalyzing the reduction of ketoacyl-CoAs to hydroxyacyl-CoAs as the substrates of PHA synthases were discovered and characterized in *Har. hispanica* [24] and *Hfx. mediterranei* [25]. Further, these enzymes displayed considerable differences to their eubacterial counterparts encoded by *phaB* genes. Only recently, Xiang summarized the genomic and enzymatic particularities of PHA biosynthesis by *Hfx. mediterranei* in a comprehensive way [26]. Figure 2 provides a simplified schematic of the pathways leading to PHB and PHBHV by eubacteria and haloarchaea, respectively. For haloarchaea, especially the multiple propionyl-CoA supplying pathways are highlighted.

## 3. *Haloferax mediterranei*—The Prototype PHA Production Strain among Haloarchaea

*Hfx. mediterranei* was the first haloarchaeon for which PHA-accumulation kinetics were studied in detail. *Hfx. mediterranei* was among the 19 organisms first isolated in 1980 by Rodriguez-Valera et al. from samples collected from the evaporation ponds of solar salterns near Alicante, Spain, and designated as strain “Q4”. In this publication, the authors reported on the isolation of “moderate and extremophile bacteria”, without discriminating between halophilic eubacteria and haloarchaea. However, this study already proposed the possibility to enrich slowly growing extremophiles from mixed microbial cultures by carrying out chemostat continuous cultivations at a low dilution rate (D) and high substrate concentration. Moreover, the authors supposed that moderate halophiles by trend prefer a rather low temperature for growth, while extreme halophiles grow best at higher temperatures. Further, this study showed for the first time that the pigmentation of extremely halophilic organisms is more pronounced at elevated temperatures, and especially so at high salinity. In their study, Rodriguez-Valera et al. described already crucial characteristics of the most outstanding among the isolates, strain “Q4”, namely negative Gram-staining, pinkish pigmentation, formation of pleomorphic rods, an optimum salinity of 250 g/L NaCl with salinity range of 100–300 g/L, and a maximum specific growth rate (µ_max._) of 0.05 1/h. Yet, this study did not search for PHA accumulation by this strain [27]. In a subsequent study, these researchers mentioned that this new isolate exhibited substantial physiological and morphological differences to other “halobacteria” described, and recommended that “R-4” (previously strain “Q4”) should be grouped into the new species *Halobacterium (Hbt.) mediterranei* [28].

In 1986, Fernandez-Castillo et al. recognized for the first time granular PHA inclusions in cells of this intriguing strain when exploring it in an experimental series with other extremely halophilic isolates (in this study termed “halobacteria”), viz. *Hbt. gibonsii*, *Hbt. halobium*, *Hbt. hispanicum*, and *Hbt. volcanii*, which were farmed in rather simple cultivation setups performed in aerated and magnetically stirred glass vessels. All these strains except *Hbt. halobium* showed PHA accumulation when growing on media containing 250 g/L salts, 10 g/L glucose, and 1 g/L yeast extract; however, *Hbt. mediterranei* by far outperformed the other strains in terms of PHA formation [29]. Today, these isolates are classified as the strains *Hfx. gibbonsii*, *Hfx. volcanii*, *Har. hispanica*, and *Har. marismortui*. These updated species names are based on a new numerical taxonomic classification based on the polar lipids of “halobacteria”; this classification was performed during the studies of Torreblanca et al.; as result, the original genus *Halobacterium* was divided into the three new genera *Haloarcula*, *Halobacterium*, and *Haloferax*; strain “*Halobacterium mediterranei*” (isolate R-4, originally “Q4”) got the new species name “*Haloferax mediterranei*” [30]. For this strain, higher PHA contents in biomass of 17 wt.% were obtained using glucose than with other substrates (citrate, cellobiose, and glycerol). Further isolates used in the study (*Hfx. volcanii*, *Hfx. gibbonsii*, and *Har. hispanica*) exhibited only minor PHA fractions in CDM of 7 wt.%, 12 wt.%, and 24 wt.%, respectively, when thriving in a medium with 250 g/L NaCl, 10 g/L glucose, and 1 g/L yeast extract. Notably, in this study, authors reported that exclusively PHB homopolyester (“poly-β-butyric acid”) was produced of by all of these strains, despite the fact that the products were subjected towards ^13^C-NMR characterization, which revealed that PHA constituents other than 3HB were present in some of the isolated PHA samples [29]. According to today´s knowledge, particularly *Hfx. mediterranei* synthetizes PHBHV copolyesters under the described cultivation conditions (sufficient supply of sugars or glycerol) [31]. Importantly, this study suggested for the first time the disruption of haloarchaeal cells by exposing them to hypotonic media (distilled water) for the facile recovery of PHA granules without the use of organic solvents, which substantially facilitates downstream processing in economic and environmental terms [29]. This study can be considered the ignition spark for setting *Hfx. mediaterranei* at the pole position of research activity with respect to PHA production by haloarchaea.

Data on the detailed exploration of PHA production by other haloarchaea are still rather scarce because, as reported by Lillo and Rodriguez-Valera [32] and Rodriguez-Valera and Lillo [31], *Hfx. mediterranei* displays higher specific growth and PHA production rates in comparison to other haloarchaea reported to accumulate PHA; this consequently is beneficial for the volumetric productivity of the bioprocess. Detailed insights into kinetics and optimized cultivation process parameters for PHA production with this strain were provided by a key publication by Lillo and Rodriguez-Valera. These authors studied continuous chemostat cultivations performed at a dilution rate D of 0.12 1/h at 38 °C. Using 20 g/L glucose and high salinity (250 g/L marine salts), 3.5 g/L PHA were produced. Replacing glucose with inexpensive starch resulted in an almost duplication of the PHA concentration (6.5 g/L), and also demonstrated the high α-amylase activity of the strain. These authors already determined that the temperature optima for growth and PHA biosynthesis by this strain are not identical [32]. Further, Antón et al. demonstrated experimentally that the organism requires highly saline nutrient media containing at least 200 g/L NaCl for optimum growth; such high salinity de facto excludes the risk of contamination with foreign germs, which is a significant gain when carrying out large-scale production setups under reduced sterility precautions [33]. This high robustness of *Hfx. mediterranei* cultivation setups against microbial rivals was later substantiated by Hermann-Krauss and colleagues, who carried out fed-batch cultivations with this strain without any sterilization provisions neither for the cultivation medium nor the bioreactor; even after several days, no infection by other microbes was detectable [34]. In contrast to high medium salinity, the cytoplasm of *Hfx. mediterranei* contains high quantities of KCl to generate high inner osmotic pressure, hence, to balance the outer osmotic pressure; this strategy of the strain to cope with such extremely high extracellular salinity [20], the so-called “salt-in” strategy, is a typical feature of haloarchaea. This requires an adaptation of the proteome, e.g., high surface charge of enzymes, to conserve the proper conformation and activity of enzymes at the edge of salt saturation. This approach drastically differs from strategies known of halophilic eubacteria, which accumulate soluble osmolytes such as ectoins as a reaction to excessive extracellular salinity [35].

PHA is not the only intriguing polymeric product produced by *Hfx. mediterranei*. In 1988, Antón et al. reported that the strain excretes also an extracellular polymer, which can be recovered from solution by precipitation with cold ethanol. This extracellular polysaccharide (EPS) causes the typical mucous appearance of *Hfx. mediterranei* colonies grown on solid nutrients [33]. This EPS is an anionic, sulfated polymer, consisting of a regular trisaccharide-repeating unit with one mannose and two 2-acetamido-2-deoxyglucuronic acid monomers and one sulfate ester bond per trisaccharide unit. Rheologically, the polymer displays xanthan-like characteristics, which has attracted interest in it as a thickening and gelling agent in food technology [36]. Later, the interrelation between parallel production and in vivo degradation of the two polymers (PHA and EPS) was investigated. It was revealed that intracellular PHA degradation is a rather slow process, even under carbon-limited conditions; technologically, this allows postponing cell inactivation and harvest after complete depletion of the carbon source without risking significant product degradation. In this study, it was also demonstrated that pronounced EPS production takes place when feeding the strain with defined carbon sources like carbohydrates, but not when supplying complex substrates like yeast extract; this trend is analogous to the strain’s PHA accumulation profile: defined carbon sources result in high PHA biosynthesis, while complex substrates favor biomass growth [37]. More details were reported by Cui et al., who studied the salinity effect on PHA and/or EPS biosynthesis as a tool to direct the carbon flux towards one product or another. These cultivations were performed in 1.2 L airlift bioreactors. In a nutshell, high salinity inhibited EPS biosynthesis, but preferred PHA accumulation. Increasing NaCl concentration from 75 g/L to 250 g/L, EPS production slightly dropped from 37 wt.% to 32 wt.%. With 71 wt.%, the PHA fraction in biomass reached its highest value at a salinity of 250 g/L NaCl; this demonstrated that a high salinity boosts PHA production at the expense of EPS formation. Technologically, these results enable a regulation of the carbon flux in *Hfx. mediterranei* by adapting the salinity of the cultivation medium in order to enhance the biosynthesis of either PHA or EPS, both constituting industrially applicable products [38]. 

**Figure 3:**
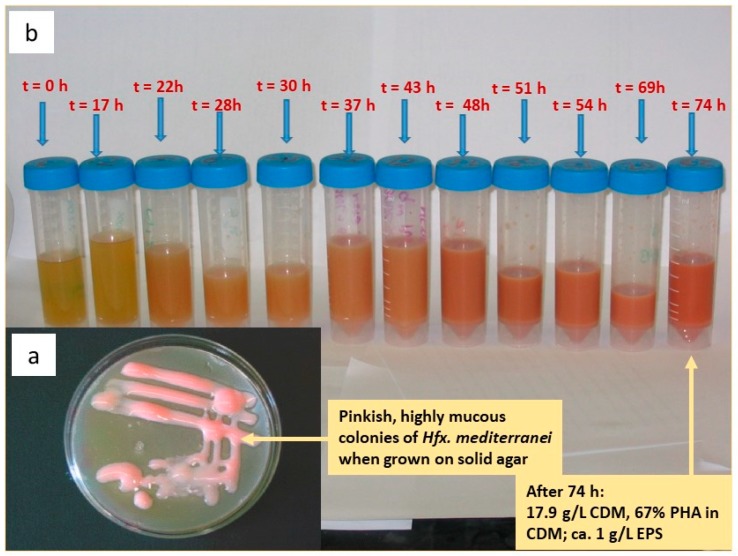
(**a**) Pinkish, mucous *Hfx. mediterranei* colonies grown on solid medium. (**b**) Macroscopic appearance of samples taken from a *Hfx. mediterranei* bioreactor cultivation from the beginning (t = 0 h) until the end (t = 74 h) of the process. Own pictures of the author M. Koller.

Figure 3a shows *Hfx. mediterranei* colonies grown on solid agar medium with yeast extract and enzymatically hydrolyzed whey permeate as substrates; the mucous and pinkish character of colonies due to the production of EPS and pigments (C50 carotenoids) is apparent. Figure 3b presents liquid samples taken from the beginning through to the end of a bioreactor cultivation of *Hfx. mediterranei* under controlled conditions in a 200 L (working volume; total volume: 300 L) pilot scale bioreactor (L 1523, Bioengineering, Wald, CH) using the same substrates: yeast extract (initial concentration 6.25 g/L) and enzymatically hydrolyzed whey permeate (initial concentration 50 g/L, corresponds to 10 g/L of an equimolar glucose/galactose mixture); a refeed of hydrolyzed whey permeate was done according to HPLC analysis of the cultivation broth after each sampling, while a re-feed of yeast extract solution was done drop-wise according to the reaction of the dissolved oxygen probe during the first phase of the cultivation (until t = 28 h) in order to provoke enhanced PHA biosynthesis. The pH-value and dissolved oxygen were permanently controlled and recorded online and PHA, EPS and protein concentrations were determined after each sampling. The samples show increasing coloration and viscosity until the end of the cultivation, when the mass fraction of PHA (a PHBHV copolyester containing 10 mol.% of 3HV amounted to 67 wt.%; final concentrations of 7.2 g/L and 1 g/L were obtained for PHBHV and EPS, respectively.

As stated above, when using simple carbon sources like carbohydrates, *Hfx. mediterranei* does not produce the homopolyester PHB as typical for the majority of wild type eubacteria, but a PHBHV copolyester. *Hfx. mediterranei* was the first strain at all, for which PHBHV copolyester production from structurally unrelated carbon sources was reported. For other strains, in vivo incorporation of 3-hydroxyvalerate (3HV) in growing PHA chains is dependent on the supply of precursors structurally related to 3HV, such as propionic acid, valeric acid, or levulinic acid. These precursors contribute considerably to the costs of PHBHV production [4]. Only decades later, the metabolic background of this particular feature was revealed by bioinformatic analysis of the *Hfx. mediterranei* genome sequence, when Han et al. proposed four active pathways in *Hfx. mediterranei*, which synthesize the 3HV-precursor propionyl-CoA. The first two pathways involve the conversion of 2-oxobutyrate (either from starting pyruvate and acetyl-CoA or starting from threonine and methionine) to propionyl-CoA. The third pathway, the so called methylmalonyl-CoA pathway, starts from the isomerization of succinyl-CoA to methylmalonyl-CoA, which gets decarboxylated to propionyl-CoA. Finally, the 3-hydroxypropionate pathway starts with carboxylation of acetyl-CoA forming malonyl-CoA, which gets reduced in a cascade of catalytic steps to propionyl-CoA. In this context, coupling of propionyl-CoA and acetyl-CoA generates 3-ketovaleryl-CoA, while condensation of two acetyl-CoA molecules, the universal, central metabolite, generates acetoacetyl-CoA. Both reactions are catalyzed by the enzyme 3-ketothioase (in older literature: β-ketothiolase). Subsequently, 3-ketovaleryl-CoA is reduced by reductases to 3HV-CoA, which acts as substrate of PHA synthases for 3HV polymerization in growing PHA chains, while acetoacetyl-CoA is transformed into 3-hydroxybuytryl-CoA, the active form of the PHB monomer 3HB [39]. Technologically important: PHBHV copolyesters, characterized by their lower crystallinity and higher difference between meting temperature and degradation temperature, are more easily processed by injection molding, melt extrusion, or other polymer processing techniques if compared with the typically highly crystalline and brittle PHB homopolyester. Moreover, due to their pronounced amorphous domains, PHBHV copolyesters are more prone to (bio)degradation in vivo and during composting if compared with PHB [40]. In particular, the PHBHV copolyester produced by *Hfx. mediterranei* typically exhibits material features desired for processing, such as a low melting temperature (T_m_), low degree of crystallinity (X_c_), high molecular mass up to the MDa range, and low polydispersity (Ð_i_), hence a high uniformity of polyester chains in one and the same sample [41].

As described, it was only during the last decade, when profound information about the enzymatic and genomic particularities of *Hfx. mediterranei*, with special emphasis dedicated to the mechanisms involved in PHA biosynthesis, was elaborated [26]. This covers studies on the special *Hfx. mediterranei* PHA synthase enzymes [18], haloarchaeal phasins as enzymes essential for PHA granule formation [42], the identification and mapping of the phaB genes encoding PHA biosynthetic enzymes in *Hfx. mediterranei* [25], the multiple pathways generating the 3HV-precursor propionyl-CoA [39,43], or patatin, the first haloarchaeal enzyme identified to serve the in vivo mobilization (depolymerization) of native *Hfx. mediterranei* granules [43].

## 4. Process Parameters for Optimized *Hfx. mediterranei*-Mediated PHA Production

In 2017, Ferre-Güell and Winterburn investigated the impact of the nitrogen sources NH_4_^+^ and NO_3_^−^ on biomass formation and PHA production by *Hfx. mediterranei*. In a N-rich medium based on glucose, yeast extract and 156 g/L NaCl, CDM and PHA content in CDM reached 10.7 g/L and 4.6 wt.%, respectively when using NH_4_^+^; with NO_3_^−^, only 5.6 g/L CDM, but 9.3 wt.% PHA in CDM were produced. Astonishingly, the type of N-source affected the composition of PHBHV copolyesters. While 16.9 mol.% 3HV were present in PHBHV when using NH_4_^+^, the 3HV fraction dropped to 12.5 mol.% when using NO_3_^−^. With NH_4_^+^, a low C/N-ratio of 42/1 resulted in reduced formation of active biomass, but increased the PHBHV share in CDM to 6.6 wt.%; the effect of the C/N-ratio was less pronounced when using NO_3_^−^. Remarkably, a lower C/N-ratio increased the 3HV share in PHBHV, which suggests an effect of the C/N-ratio on the activity of the propionyl-CoA generating pathways. Interestingly, no 3HV was detected in PHA before the polyester concentration reached 0.45 g/L. Hence, detailed understanding of the effect of type and concentration of different N-sources can contribute to the enhanced production of PHBHV copolyesters by haloarchaea with pre-defined composition and characteristics [44]. As a follow-up study, Melanie and colleagues examined the impact of the initial phosphate concentration on PHA production by *Hfx. mediterranei*; 0.95 g/L PHBHV (15.6% in CDM) with unexpectedly high 3HV content (22.36 mol.%) was produced after seven days of cultivation with 156 g/L NaCl and 0.5 g/L KH_2_PO_4_ as P-source in 500 mL shaking flasks. Lower initial KH_2_PO_4_ concentrations (0.25 or 0.00375 g/L) caused lower PHA productivity and lower 3HV fractions in PHBHV copolyesters. Thermal characterization of the products revealed data typical for PHA produced by *Hfx. mediterranei* [45]. Moreover, Cui et al. studied the temperature effect on biomass and PHA formation by *Hfx. mediterranei*. This was done by developing, calibrating, and validating a mathematical model for growth and PHA production kinetics at 15, 20, 25, and 35 °C. The kinetic coefficients implemented into the model were obtained by experimental results from cultivations carried out in stirred and aerated flasks in a medium of similar composition to molasses wastewater. As a result, it was shown that the cultivation temperature considerably effects PHA production by *Hfx. mediterranei*; at 15 °C, the volumetric PHA productivity amounted to only 390 mg/(L·h), while 620 mg/(L·h) were obtained in cultivations at 35 °C. An Arrhenius equation plot was drawn that revealed the maximum specific growth rate (µ_max._; 0.009 1/h at 15 °C, 0.033 1/h at 35 °C), maximum specific substrate uptake rate (q_Smax._; 0.018 g/(g·h) at 15°C, 0.037 g/(g·h) at 35 °C), and specific decay rate (k_d_; 0.0048 1/h at 15 °C, 0.0089 1/h at 35 °C) were higher at increased temperature. The calculated activation energy for biomass growth, decay, and substrate uptake were 58.31 kJ/mol, 22.38 kJ/mol, and 25.59 kJ/mol, respectively. For all investigated temperatures, the developed model was of high predictive power. Even with sufficient supply with nitrogen source, the elevated temperature level of 35 °C significantly improved PHA productivity; this temperature was therefore recommended as the optimal cultivation temperature to be used for this strain. Furthermore, the 3HV fraction in PHBHV turned out to be independent from temperature; under all temperature conditions, the PHBHV copolyesters contained 16.7 mol.% 3HV. These data are of high importance, because information in older literature for the temperature optimum of *Hfx. mediterranei* were inconsistent and even contradictory [46]. Unfortunately, the authors did not study higher temperatures, which were reported in the basic publications for *Hfx. mediterranei* as optimum for growth and PHA-biosynthesis (50 °C and 45 °C, respectively) [32].

## 5. Use of Different Feedstocks for PHA Biosynthesis by *Hfx. mediterranei*

A range of diverse inexpensive carbon-rich food and agro-industrial waste and side products have already been tested as feedstocks for PHA production by *Hfx. mediterranei*. This encompasses surplus whey from cheese and the dairy industry [41,47,48], crude glycerol phase (CGP) as the main by-product of biodiesel production [33], extruded corn starch [49], extruded rice bran [50], stillage from bioethanol manufacturing [51,52], molasses wastewater form sugar industry [48], olive mills wastewater [53], vinasse from molasses-based ethanol production [54], or macroalgae (seaweeds) hydrolyzed by advanced techniques [55].

### 5.1. Hfx. mediterranei on Hydrolyzed Whey Permeate

Indeed, *Hfx. mediterranei* is considered one of the most auspicious organisms for whey-based PHA production on a large scale because of its high production rates, high robustness and the stability of fermentation batches, as well as convenient product recovery via hypo-osmotic cell disintegration [41]. The strain grows excellently on both acidic or enzymatically hydrolyzed whey permeate (equimolar mixtures of glucose and galactose as main carbon source; permeate generally separated from whey retentate via ultrafiltration) but does not utilize intact lactose [56]. In bioreactor cultivation setups, high maximum specific growth rates (µ_max._) of 0.11 1/h were reported when using hydrolyzed whey permeate as a substrate; this is substantially higher than specific growth values reported for other haloarchaea. Maximum values for specific PHA production (q_P_) amounted to 0.08 g/(g·h). When optimizing the cultivation conditions (inoculum preparation, medium composition), these values were even enhanced to µ_max._ = 0.09 g/(L·h) and q_P_ = 0.15 g/(g·h), respectively. Biomass concentration and PHA fractions in biomass reached 16.8 g/L and 73 wt.%, respectively [57]. Importantly, when cultivated on hydrolyzed whey lactose (equimolar mixture of glucose and galactose), *Hfx. mediterranei* has a clear preference for glucose, which results in the accumulation of galactose in the fermentation medium, thus drastically increasing the biochemical oxygen demand of spent fermentation broth and causing the loss of a substantial part of the substrate. Suggested solutions to solve this ecological and economic shortcoming involve separating galactose from spent fermentation broth for further use, e.g., as a sweetener or nutritional and pharmaceutical additive; yet, this approach is economically rather doubtful. In this context, Pais et al. discovered that the activity of the strain´s enzymes involved in galactose conversion can be increased by adaptation of the trace elements supply to the cultivation medium; this way, a more complete substrate conversion was achieved. Remarkably, the PHBHV copolyesters obtained in this study had a very low 3HV fraction of only 2 mol.% 3HV [48].

Attempts to further increase the material features of whey-based PHA produced by *Hfx. mediterranei*, the precursor substrates valeric acid and γ-butyrolactone (GBL) were supplied in bioreactor cultivations with 200 g/L NaCl and hydrolyzed whey permeate as main carbon source. These precursors were added in order to achieve higher 3HV fraction in PHA, and to introduce 4HB as an additional PHA building block. This way, a poly(3HB-*co*-21.8%-3HV-*co*-5.1%-4HB) terpolyester with encouraging material properties (low melting points, high molecular mass and low crystallinity) was produced, and suggested for further use in the medical field [47].

It is obvious that the highly saline waste streams of *Hfx. mediterranei* cultivations need appropriate handling, hence sustainable disposal or re-utilization in order to reduce process costs and to minimize the risk of environmental pollution. Importantly, disposing salt-rich materials after cell harvest and PHA recovery constitutes a real environmental threat, especially for large cultivation setups; the concentration of total dissolved solids (TDS) in disposed wastewater is limited with 2 g/L according to valid environmental norms. Therefore, the possibility of re-using saline cell debris, which remains after PHA recovery, as well as recycling the salt-rich spent fermentation broth by using it as mineral source in new cultivation setups was studied. Experiments with spent fermentation broth and saline cell debris were carried out; the results underlined the viability of recycling these waste streams. It was demonstrated that re-using spent fermentation broth for the preparation of new saline mineral medium drastically reduces the need for fresh salts. Furthermore, substituting up to 29% of yeast extract, typically a costly component used in *Hfx. mediterranei* cultivation media, for saline PHA-free cell debris gave growth rates similar to those obtained in the original cultivations [58].

Data from fed-batch cultivations on 200 L pilot scale, which are as yet the only results for large-scale PHA production using haloarchaea and inexpensive feedstock, were used for the cost assessment of PHBHV production on hydrolyzed whey by *Hfx. mediterranei*. This assessment encompassed the profits of solvent-free PHA recovery in distilled water, inexpensive acidic whey hydrolysis by mineral acids, abandoning any sterility provisions, copolyester production without the need for 3HV precursors, and the re-use of saline cell debris and spent fermentation broth in subsequent fermentation batches. A price of less than € 3 was estimated for the production of 1 kg PHA, which is significantly less than is typically reported PHA production prices of 5–10 €/kg. This pilot scale calculation delivered 7.2 g/L PHA and a volumetric productivity of 0.11 g/(L·h). The study also compared value creation for converting whey to, on the one hand, PHA, with, on the other hand, to whey powder, the currently most common application. In addition to cost assessment, a life cycle assessment (LCA) using the “sustainable process index” (SPI) as indicator for sustainability was carried out. A significant result of this process suggested that the estimated ecological footprint of whey-based PHA produced by *Hfx. mediterranei* is superior to fossil-based plastics if all process side streams are recycled [59]. This is in accordance with more recent considerations published by Narodoslawsky and colleagues, who concluded that the ecological footprint of “bioplastics” outperforms established plastics only when considering and optimizing the entire life cycle of the polymer [60].

### 5.2. Hfx. mediterranei on Crude Glycerol Phase from Biodiesel Industry

Beside hydrolyzed whey, strain *Hfx. mediterranei* accumulates PHA heteropolyesters (co- and terpolyesters) also when being fed with crude glycerol phase (CGP). CGP constitutes the main side-product of biodiesel production, which is steadily emerging in many global areas. In this context, CGP was used as feedstock for fed-batch bioreactor cultivations of *Hfx. mediterranei* in media containing 150 g/L NaCl; in these experiments, a volumetric PHBHV productivity of 0.12 g/(L·h) and a product fraction of 75 wt.% PHBHV (10 mol.% 3HV) in CDM were reached. Co-feeding the 4HB-precursor GBL together with the main substrate CGP, a PHA terpolyester containing 3HB (83 mol.%), 3HV (12 mol.%), and 4HB (5 mol.%) was synthesized [32]. Here, it should be added that the utilization of glycerol for PHA biosynthesis is not a typical feature for other haloarchaea; many of them use this substrate for the production of non-PHA biomass and maintenance energy, but not for PHA biosynthesis [61].

### 5.3. Hfx. mediterranei on Processed Starchy Materials

Extruded rice bran (ERB) and extruded cornstarch (ECS) were applied by Huang et al. as additional inexpensive substrates for PHA production by *Hfx. mediterranei*. For this purpose, 5 L scale bioreactor cultivations were performed in repeated fed-batch mode under pH-stat conditions with a medium containing 234 g/L NaCl and all other compounds required by the strain. Due to the insufficient utilization of non-processed ERB and ESC, these feedstocks were extruded before being supplied as substrate as ERB/ECS mixtures in a ratio of 1/8 (g/g). High values for CDM, PHA, and PHA content in biomass were reported: 140 g/L, 77.8 g/L, and 56 wt.%, respectively [51]. In a similar way, Chen and colleagues used cornstarch treated by an enzymatic (α-amylase) reactive extrusion process for PHA production by *Hfx. mediterranei*. The cultivation was performed in a 6 L bioreactor under pH-stat fed-batch cultivation conditions and a salinity of 234 g/L NaCl. Carbon and nitrogen concentration in the cultivation broth was kept constant by feeding a stream containing a 1/1.7 (g/g) mixture of extruded ECS (carbon source) and yeast extract (nitrogen source). After 70 h, the PHA concentration and PHA content in CDM reached 20 g/L and 51 wt.%, respectively. Similar to other PHA production setups carried out with *Hfx. mediterranei*, a PHBHV copolyester with 10.4 mol.% 3HV was produced by the strain without supply with 3HV precursor compounds. This process reached the as yet highest volumetric PHA productivity with *Hfx. mediterranei* with around 0.28 g/(L·h) [49].

### 5.4. Hfx. mediterranei on Waste Streams of Bioethanol Production

Vinasse constitutes a recalcitrant waste of ethanol production based on molasses. On shaking the flask scale, this waste product was studied as a potential substrate for PHA production by *Hfx. mediterranei*. Pre-treatment by adsorption on charcoal was carried out to remove inhibiting compounds from vinasse, mainly phenolic compounds. Using 25–50% (v/v) pre-treated vinasse delivered a maximum PHA content in biomass of 70 wt.%, a maximum PHA concentration of 19.7 g/L, a volumetric productivity for PHA of 0.21 g/(L·h), and a substrate conversion yield of 0.87 g/g. By this process, about 80% of the (bio)chemical oxygen demand of pre-treated vinasse were removed. Further, in these experiments PHA was recovered from biomass by cell lysis in a hypotonic medium and further purified by treatment with sodium hypochlorite and organic solvent. Again, the product was characterized as a PHBHV copolyester. Using 25% pre-treated vinasse, the 3HV fraction in PHBHV amounted to 12.4 mol.% and increased to 14.1 mol.% when using 50% pre-treated vinasse. The high medium salinity allowed the performance of the cultivation without prior sterilization of the bioreactor and medium, which contributes to production cost reduction. The authors underlined that the simple use of charcoal for vinasse detoxification was more economical than the above described processes using other waste materials as substrates; ultrafiltration to concentrate whey, or extrusion and enzymatic treatment of starchy materials contribute more to the production cost than charcoal pre-treatment. Analogous to the processes based on whey, which can be integrated into the production lines of dairies and cheese factories, where whey directly accrues as waste stream, the vinasse-based process can easily be integrated into distilleries, where it even contributes to the treatment of process wastewater [54].

As the main waste material stemming from ethanol manufacture based on rice, raw stillage was applied without pre-treatment by the same team of researchers as another inexpensive substrate for PHA biosynthesis by *Hfx. mediterranei*. These experiments strongly focused at closing material cycles within the process, and to reduce its environmental impact; for this purpose, medium salts from previous cultivation batches were directly recycled. 16.4 g/L PHA, about 70 wt.% PHA in biomass, a substrate conversion yield of 0.35 g/g, and a volumetric PHA productivity of 0.17 g/(L·h) were achieved in shaking flask experiments. In analogy to above described experiments performed using whey, a PHBHV copolyester with 15.3 mol.% 3HV was produced. A reduction of the (bio)chemical oxygen demand in feedstock stillage by about 85%, and a decrease of total dissolved solids (TDS) in spent fermentation broth to only 0.67 g/L were reached [50].

As follow up study, Bhattacharyya et al. performed a techno-economic assessment of *Hfx. mediterranei*-mediated PHA production on unsterile waste stillage from rice-based ethanol manufacturing. This process was performed in a plug-flow bioreactor made of plastic, which is normally used to study and optimize activated sludge processes. *Hfx. mediterranei* successfully utilized stillage, and produced 63 wt.% PHA in biomass, while PHA concentration, product yield, and volumetric productivity amounted to 13 g/L, 0.27 g/g, and 0.14 g/(L·h), respectively. A significant reduction of the (bio)chemical oxygen demand of stillage by 82% was reached. The accumulated PHA was identified as PHBHV copolyester with a molar 3HV fraction of 18%. An innovative desalination process of the supernatant of spent fermentation broth, consisting of two steps, was developed; this process involved stirring and heating the spent supernatant with decanoic acid. After cooling and settling, the mixture separated into three phases: salts precipitated and became available for subsequent fermentation batches, an organic phase of lower density (decanoic acid; to be applied for subsequent desalination cycles), and the heavier water phase. By this simple approach, it was possible to recover 99.3% of the medium salts and to re-use them in next PHA production batches. An assessment of cost for PHBHV produced via this process, which was suggested by the authors as the basis for the design of a pilot plant, estimated US $ 2.05 per kg of product; this calculation refers to a production plant with a production capacity of 1890 annual tons. It is important to note that desalination particularly contributed considerably to this low-cost estimate. Further, this techno-economic analysis holds promise for the realization of PHA production integrated in existing industrial production plants, in the case of rice-based stillage especially in emerging countries like India [52].

### 5.5. Hfx. mediterranei on Wastewater of Olive Oil Production

Using olive oil wastewater (OMW), a highly contaminated side stream of the olive processing industry, *Hfx. mediterranei* was cultivated in a one-stage cultivation process aiming at PHBHV production. In this process presented by Alsafadi and Al-Mashaqbeh, the inexpensive feedstock OMW was supplied to the culture without pre-treatment, which saved costly steps, e.g., for dephenolization. When the entire cultivation medium contained up to 25% OMW, the present phenolic compounds did not inhibit grow of the strain. The cultivation conditions were optimized to achieve maximum polymer yield and PHA fraction in biomass; this encompassed fine-tuning salinity, temperature, and oxygen supply. A salt concentration of 220 g/L NaCl and a temperature of 37 °C turned out to be best values for optimum PHA productivity. The accumulated biopolyester was recovered from biomass by hypotonic cell lysis assisted by SDS and vortexing, and further purified with sodium hypochlorite, thus using only minor amounts of organic solvents. The relative content of 3HV in the generated PHBHV copolyester amounted to 6%, which is significantly lower than 3HV fraction for *Hfx. mediterranei* PHBHV produced on other substrates as described above for whey, stillage, starchy materials, or vinasse. This process was suggested by the authors to enhance OMW valorization, and to reduce production cost of desired bioproducts. However, taking into account that the cultivations were performed only on a small shaking flask scale, upscaling to the bioreactor scale, to be carried out under controlled cultivation conditions, it will be necessary to assess industrial viability [53].

### 5.6. Hfx. mediterranei on Hydrolyzed Macroalgae

The green seaweed (microalga) *Ulva* sp., an organism typically producing unwanted algal blooms at coastal areas, was hydrolyzed by alkaline (4.8 mM KOH) and thermal (180 °C) batch treatment and used as substrate for shaking flask cultivations of *Hfx. mediterranei* at 42 °C and pH 7.2. PHA concentration, CDM, PHA content in biomass and 3HV fraction in PHA obtained when using 25% of Ulva hydrolysate reached 2.2 g/L, 3.8 g/L, 58 wt.%, and 0.08 mol/mol, respectively [55].

## 6. Microstructure of *Hfx. mediterranei* PHA Copolyesters

*Hfx. mediterranei* produces PHBHV copolyesters, which are not homogenous materials, but consist of diverse fractions of varying molecular mass and monomeric composition. PHBHV produced by *Hfx. mediterranei* using glucose and yeast extract was separated into two fractions with different 3HV contents by using a mixture of chloroform and acetone. The predominant fraction, which amounts to about 93% of the entire polymer had a 3HV fraction of 10.7 mol.% and a molecular mass of about 570 kDa, while the minor fraction contained considerably lower amounts of 3HV (12.3 mol.%) and had a significantly lower molecular mass of 78.2 kDa. This low-molecular mass fraction was soluble even in acetone, which typically is reported as an “anti-solvent” for short-chain-length PHA like PHB or PHBHV at temperatures below the boiling point of the solvent. T_m_ and T_g_ of both fractions were similar, and both had rather low Ð_i_ values. By DSC characterization at heating rates below 20 °C/min, two overlapping melting peaks became visible in the DSC traces, with varying relative peak intensities when changing the heating rate; the authors supposed that this effect might originate from melt-and-recrystallization phenomena in PHA [62]. In another study, a low-molecular mass (209 kDa) fraction of a *Hfx. mediterranei* poly(3-hydroxybutyrate-*co*-3-hydroxyvalerate-*co*-4-hydroxybutyrate) terpolyester was extracted by acetone under reflux conditions in a Soxleth apparatus, while the major part of the product amounting to about 99%, which had a considerably higher molecular mass exceeding 1 MDa, was soluble in acetone only at a temperature exceeding acetone´s boiling point [63]. Both studies confirmed the presence of intracellular PHA blends in *Hfx. mediterranei*. More detailed insights into the microstructure of PHBHV produced by *Hfx. mediterranei* were disclosed by Han et al., who described the complex blocky structure of the biopolyester (*b*-PHA), which consists of alternating PHB and poly(3-hydroxyvalerate) (PHV) blocks, which are linked to blocks of randomly distributed PHBHV copolyesters. These researchers also demonstrated that *b*-PHA production by *Hfx. mediterranei* could be fine-tuned via the co-feeding of glucose and valerate. Because of this “blocky” structure and its high 3HV content, *Hfx. mediterranei b*-PHA displays exciting material features such as low degree of crystallinity and improved Young’s modulus. Films of this polyester showed unique foveolar cluster-like surface morphology with high roughness. This enables a possible biomedical application of this *b*-PHA, as revealed by its better blood platelet adhesion and faster blood clotting behavior in comparison to randomly distributed PHBHV [64].

A fed-batch process using mixtures of butyric and valeric acid as substrate for *Hfx. mediterranei* was described by Ferre-Güell and Winterburn; this process was designed to synthesize PHBHV copolyesters with pre-defined composition in a reproducible way. Tween 80 added as emulsifier at a temperature of 37 °C improved the bioavailability of the substrates; the highest PHBV contents in biomass (59 wt.%) and volumetric productivity (10.2 mg/(L·h)) were reported for a butyric/valeric acid mix of 56/44. The biopolyester had a pre-defined 3HV fraction of 43 mol.%. This triggering of the PHBHV composition by adapting the composition of the substrate mix was realized both on shaking flask and bioreactor scale under different temperatures and emulsifier concentrations. Only insignificant variances in PHBHV product quality (molecular mass, thermo-mechanical properties) were observed for different production scales (bioreactor or shaking flask, respectively), which demonstrated the convenient scalability of this process [65]. A similar study aimed also at manufacturing *Hfx. mediterranei* PHBHV copolyesters of controlled composition and microstructure. Both *b*-PHBHV and PHBHV of random distribution were produced by supplying cultures with different fatty acids with an even (acetic, butyric, hexanoic, octatonic, and decanoic acid) or an odd number (propionic, valeric, heptanoic, nonanoic, undecanoic acid) of carbon atoms. Only those fatty acids with less than seven carbon atoms were accepted by the strain as a substrate for growth and PHA production. When feeding acetic acid, a PHBHV copolyester with about 10 mol.% 3HV was produced, which is in accordance to the 3HV fraction typically obtained when using glucose, glycerol, etc., while in the case of butyric acid, almost no 3HV was found in the copolyesters. Valeric acid used as sole carbon source resulted in an exceptionally high 3HV content of more than 90 mol.%. When using propionic acid, the 3HV content was lower than in the case of valeric because of the partial oxidative propionyl-CoA decarboxylation, which converts propionyl-CoA to CO_2_ and acetyl-CoA, which acts as 3HB precursor. Applying different feeding strategies for butyric acid, valeric acid and mixtures of these acids, it was shown that sequential feeding creates *b*-PHBHV containing alternating PHB and PHV blocks, while random distribution of 3HB and 3HV occurs when co-feeding the substrates. Furthermore, higher 3HV fractions in randomly distributed PHBHV resulted in higher PHA chain mobility in the amorphous phase of the polyesters, which was evidenced by lower T_g_ values. In general, higher 3HV fractions in random PHBHV resulted in decreased polyester crystallinity, lower T_m_, improved ductility, and higher elasticity, which consequently enhances processibility of the polymers [66].

## 7. Further Haloarchaeal Genera Encompassing PHA Producers

### 7.1. Haloarcula *sp*.

Beside the broadly described strain *Hfx. mediterranei*, other haloarchaea with more or less pronounced PHA production capacity were isolated from different saline environments. In this context, Altekar and Rajagopalan exposed the interrelation between PHA accumulation by the haloarchaea *Hfx. mediterranei*, *Hfx. volcanii* and *Har. marismortui*, and CO_2_-fixation activity catalyzed by ribulose bisphosphate carboxylase (RuBisCo) present in the cell extracts of these strains [67]. Later, Nicolaus and colleagues isolated three previously unknown organisms from Tunesian marine salterns. These isolates grew well under extremely saline conditions (3.5 M NaCl, ~200 g/L). All three strains displayed parallel PHB production and EPS excretion when supplied with different carbon sources. By analyzing the strains´ lipid patterns, it was concluded that all of them belong to the genus *Haloarcula* (*Har.*). All of them grew on starch, and one (isolate “T5”) grew expediently on the inexpensive substrate molasses. After growing about ten days on starch or glucose, this strain T5 accumulated 0.5 wt.% PHB in CDM, and 1 wt.% when growing on molasses. DNA-DNA hybridization tests and other biochemical studies identified strain T5 as a new *Har. japonica* ssp. [11]. Legat and colleagues later revealed by means of Nile Blue and Sudan Black staining and ^1^H-NMR investigation of freeze-dried cells that also *Har. hispanica* strain DSM 4426^T^ constitutes a producer of PHA, more precisely of PHBHV copolyesters [68].

*Haloarcula* sp. IRU1 was isolated from the hypersaline Iranian Urmia lake. In shaking flask cultivations, PHB production was optimized by using varying carbon, nitrogen, and phosphate source concentrations and by studying the effect of temperature in between 37 °C and 55 °C. Highest PHB contents in biomass of 63 wt.% were reported for 42 °C when using 2 g/L glucose, 0.2 g/L NH_4_Cl, and 0.004 g/L KH_2_PO_4_ [69]. Later, glucose, fructose, sucrose, starch, acetate, and palmitic acid were tested as substrates for *Har.* sp. IRU1. Using glucose, CDM and PHB concentration substantially increased compared to all other carbon sources, while lowest CDM and PHB concentration were reported for acetate and palmitic acid [70]. The same organism, *Har.* sp. IRU1, was also cultivated on a medium containing petrochemical wastewater as carbon source; as determined by a Taguchi experimental design, highest PHB fractions in biomass (47 wt.%) were obtained with 2% wastewater, 0.8% tryptone, 0.001% KH_2_PO_4_ and a temperature of 47 °C [71]. In addition, *Har.* sp. IRU1 was also cultivated in minimal media containing crude oil as sole carbon source; axenic cultivations for five days with 2% crude oil, 0.4% yeast extract, and 0.016% NaH_2_PO_4_ at 47 °C were carried out. Unfortunately, only the highest PHB fraction in CDM (41 wt.%) was reported in this article, but no data for productivity or PHB concentration were reported. Still, *Har.* sp. IRU1 was proposed as promising organism for bioremediation of petrochemically polluted environments, combined with value-added PHB biosynthesis [72]. Finally, even textile wastewater was investigated as a substrate for this strain to thrive; in this study, PHA biosynthesis was not monitored [73].

Vinasse, a side-product of molasses-based ethanol manufacturing, comprises non-volatile phenolic compounds, which remain in the residue after distillative ethanol removal; these phenolic compounds are known inhibitors of microbial growth. In 2012, vinasse was studied by Pramanik and colleagues as a substrate to cultivate the extremely halophile *Har. marismortui* [74], the first haloarchaeon unambiguously shown to produce PHB by Kirk and Ginzburg already in the early 1970ies [10]. Using a highly saline (200 g/L NaCl) cultivation medium containing 10% raw vinasse, *Har. marismortui* accumulated 26 wt.% PHB in CDM and reached a volumetric PHB productivity of 0.015 g/(L·h) in shaking flask experiments. These values became considerably better after removing the phenolic compounds by well-established absorption on charcoal; in a medium consisting of 100% dephenolized vinasse, 30 wt.% PHB in CDM and a volumetric productivity of 0.02 g/(L·h) were reached [66]. However, it should be noticed that PHA biosynthesis is not observed in all *Har.* sp.; e.g., Oren and colleagues did not detect any PHA inclusions when investigating the red, square-shaped Egyptian brine-pool isolate *Har. quadrata* in details [75].

### 7.2. Halogeometricum *sp.*

In 2013, Salgaonkar et al. screened seven extremely halophilic Archaea isolated from brine and sediments of solar salterns in India. Defined saline cultivation media with 200 g/L NaCl turned out to be suitable for the growth of all seven microbial isolates; all of them also accumulated PHA. Based on phenotypic and genotypic tests, six strains out of them were grouped into the genus *Haloferax*, and named as strains TN4, TN5, TN6, TN7, TN10, and BBK2, while isolate TN9 was described as the new taxonomic species *Halogeometricum (Hgm.) borinquense*. This new organism performed most auspiciously among the seven isolates, and was investigated in more detail regarding its growth and PHA accumulation kinetics. It was revealed that highest PHA accumulation rates for strain *Hgm. borinquense* TN9 already take place during the exponential phase of growth, hence prior to depletion of growth-essential nutrients. This “growth-associated PHA-production” characteristic differs from most other reported PHA production strains, which typically display maximum PHA productivity not before nutrient deprivation. In biomass, 14 wt.% PHB homopolyester was produced after a cultivation period of five days [76]. Later, the same researchers isolated another haloarchaeon from the Marakkanam solar salterns in Tamil Nadu, India. This new organism was labeled *Hgm. borinquense* E3; the strain produced PHBHV copolyesters when growing in a highly saline medium on glucose as the sole carbon substrate. This copolyester production is similar to the findings discussed above for *Hfx. mediterranei*, but in contrast to the strain´s close relative *Hgm. borinquense* TN9, a strain which produced only PHB homopolyester on glucose. Shaking flask cultivation experiments lasting four days resulted in a high intracellular polymer content of 74 wt.% PHBHV (with 22 mol.% 3HV) in biomass [77]. Additionally, the same research team cultivated four wildtype haloarchaea on hydrolyzed sugarcane bagasse (hSCB), which constitutes an amply available by-product of sugar manufacturing mainly consisting of lignocelluloses. Among these organisms, *Hgm. borinquense* E3 exhibited the highest PHA productivity according to fluorescence measurements after Nile Red staining. The organisms were identified as *Haloferax volcanii* BBK2 (one of the strains isolated in [70]), *Haloarcula japonica* BS2, and *Halococcus salifodinae* BK6. As also described for *Hfx. mediterranei*, strain *Hgm. borinquense* E3 forms slightly pink colored colonies with a slimy appearance, which demonstrated pigment and EPS biosynthesis. In a medium containing 200 g/L NaCl at 37°C and 25% or 50% hSCB, *Hgm. borinquense* E3 was cultivated for six days in shaking flasks. The PHA fractions in biomass amounted to 50 wt.% (25% SCB) and 46 wt.% (50% hSBC), respectively, while specific production rates (q_p_) were reported with 3.0 mg/(g·h) for 25% hSCB, and with 2.7 mg/(g·h) for 50% hSBC. A PHBHV copolyester with 13.3 mol.% 3HV was isolated from biomass [78]. Subsequent studies with *Hgm. borinquense* E3 resorted to starch-based waste materials used as substrates for PHA production. In this context, pure starch and acid-hydrolyzed cassava waste were used in parallel shaking flask cultivation experiments at a salinity of 200 g/L NaCl. After ten days, 4.6 g/L PHBHV (13.1 mol.% 3HV) were produced on pure starch, while the use of cassava waste delivered 1.5 g/L PHBHV (19.7 mol.% 3HV) [79]. It is noteworthy that, unfortunately, all cultivations with *Hgm. borinquense* were carried out on a shaking flask scale; scale up experiments under controlled conditions in bioreactors are still missing in the literature.

### 7.3. Halopiger *sp.*

A corrosion-resistant bioreactor consisting of polyether ether ketone (PEEK), tech glass and silicium nitrite ceramics was constructed by Hezayen and colleagues [80]. This new composite bioreactor was used for the cultivation of two new extremely halophilic isolates. One of them, “strain 56”, today known as *Halopiger (Hpg.) aswanensis* DSM 13151, was studied for PHB production in a medium containing more than 200 g/L NaCl. The other strain, *Natrialba* (*Nab.*) sp., was used to synthesize poly(γ-glutamic acid) as target product in a medium of the same salinity. Both organisms were isolated from hypersaline samples taken from the soil of the Egyptian city Aswan. PHB production by “strain 56” (*Hpg. aswanensis*) on acetate and n-butyric acid as mixed substrate amounted to 4.6 g/L, and the PHB content in CDM to 53 wt.% after 12 days cultivation in batch-mode. It was determined that 40 °C was the optimal temperature to cultivate this strain. The isolated biopolyester had a M_w_ of 230,000 g/mol and a Ð_i_ of about 1.4 [80]. In a follow-up study, Hezayen et al. reported for the first time on a PHA synthase of haloarchaea; here, the authors investigated crude extracts of “strain 56” in environments supporting PHA biosynthesis. A protocol for release of PHA granules by cell lysis in hypotonic medium and separation of granules by differential centrifugation was developed, and the granule-associated PHA synthase was studied and characterized [14]. Later, this strain, which forms Gram-negative, motile, pleomorphic pink rods, was biochemically and taxonomically categorized, and is nowadays known as *Hpg. aswanensis* DSM 13151. The organism was reported to produce large amounts of PHB; it also excretes an EPS, which causes high viscosity of the cultivation broth. High salinity of 220–250 g/L NaCl, pH-value 7.5 (range: 6–9.2) and a temperature of 40 °C (maximum accepted temperature: 55 °C) were determined as the optimum condition for this extreme halophilic species to thrive [81].

### 7.4. Halobiforma *sp.*

In the study published by Hezayen and colleagues [14], another red pigmented (carotenoid-rich) aerobic organism was isolated from hypersaline Egyptian soil in Aswan. When cultivated for eight days in shaking flasks on butyric acid, this “strain 135^T^” accumulated up to 40 wt.% PHB in biomass; on complex substrates like casamino acids, peptone, or yeast extract, even 15 wt.% PHB in biomass were accumulated. This organism requires at least 130 g/L NaCl for biomass growth, and a temperature of 42 °C revealed best growth. The authors classified the new isolate as species *Halobiforma (Hbf.) haloterrestris* sp. nov. (DSM 13078^T^) [82]. *Hbf. lacisalsi* sp. nov., a close microbial relative from the genus *Halobiforma*, was later isolated by Xu and associates from a salt lake in China. This organism was shown to grow optimally at 100 g/L NaCl; unfortunately, no tests were reported that refer to PHA biosynthesis [83].

### 7.5. Natrinema *sp.*

Danis et al. investigated five extremely halophilic archaeal isolates in order to identify new extremophilic strains with high capacity for PHA biosynthesis; the conversion of different inexpensive raw materials such as cornstarch, melon, apple, and tomato processing waste, sucrose, and whey. Among these materials, cornstarch appeared as the most encouraging substrate for PHA biosynthesis, while among the five isolated haloarchaea, strain 1KYS1 showed highest PHA production capacity. Via comparative 16S rRNA gene sequence analysis, it was revealed that strain 1KYS1 was closely related to the extremely halophilic genus *Natrinema* (*Nnm.*), and, within this genus, to the strain *Nnm. pallidum* JCM 8980. When cultivated on starch as single carbon source and a salinity of 250 g/L NaCl, strain 1KYS1 accumulated 0.53 g PHA per g of its biomass. Transmission electron microscopy (TEM) revealed that the accumulated material, a PHBHV copolyester, forms large, uniform granules (“carbonosomes”), which, after cell lysis, is a considerable benefit for the convenient separation of PHA granules via floatation or centrifugation. In addition, this biopolyester was blended with low molar mass poly(ethylene glycol), which resulted in the preparation of a new type of biocompatible polymer film, which has been applied for drug release studies using the antibiotic Rifampicin [84]. In 2018, the haloarchaeon *Natrinema ajinwuensis* RM-G10 (synonym: *Natrinema altunense* strain RM-G10) was isolated from salt production pans in India. *Nnm. ajinwuensis* accumulated about 61 wt.% PHA in biomass and showed high volumetric PHA productivity of 0.21 g/(L·h) when cultivated for 72 h in repeated batch shaking flask cultivation setups on glucose. Using glycerol instead of glucose resulted in biomass formation, but not in PHA biosynthesis. The product based on glucose turned out to be a PHBHV copolyester with a 3HV fraction in PHBHV of 0.14 mol/mol, which is a value similar to those reported for other haloarchaeal strains (*vide supra*). When analyzed by DSC, the biopolyesters showed two separated melting endotherms (T_m_ 143 °C and 157.5 °C), T_g_ of −12.3 °C, an onset of decomposition temperature (T_d_) of 284 °C, and a degree of crystallinity (X_c_) of 35.45%. 200 g/L NaCl were reported as optimal salinity for both biomass growth and PHA production by this organism [61].

### 7.6. Haloquadratum *sp.*

Unusual organisms, originally isolated at the Egyptian Sinai Peninsula, were described in 1980 by Walsby, who was interested in the highly refractive gas vesicles produced by the microbes; this researcher described his isolates as “ultra-thin square bacteria” [85]. A quarter of a century later, Walsby reported that cells of this strain resemble “thin, square or rectangular sheets with sharp corners”, and reported their dimensions being 2–5 μm wide but not even 0.2 μm thick. The outstanding low thickness of sheets makes them bulge slightly, with gas vesicles visible along their edges; he also noted that this organism thrives “at the edge of water activity”. Importantly, Walsh also observed “poly-β-hydroxybutyrate granules in the corners” [86]. The organism was for a long time believed to be not culturable in monoseptic cultures, and its genome was deciphered not before 2006 by Bolhuis et al.; these researchers revealed that the strain´s genome encodes photoactive retinal proteins of the membrane and S-layer glycoproteins of the cell wall. In this study, the species name *Haloquadratum (Hqr.) walsbyi* was used [16]. Later, Burns et al. investigated two closely related novel square-shaped aerobic, extremely halophilic members of the haloarchaea, isolated from saltern crystallizers in Australia and Spain, and classified both of them as members of the new species *Hqr. walsbyi*. In this study, the authors described that growth of this occurs at pH 6.0–8.5, 25–45 °C and 14–360 g/L NaCl. The extremely halophilic cells lyse immediately in distilled water and a minimum of ~140 g/L salts is required for growth. Optimal growth occurs under neutral to alkaline conditions, above 180 g/L NaCl. By electron cryomicroscopy, PHA inclusions were reported by the authors, but not further studied or quantified [87]. Nile Blue A and Sudan Black staining of *Hqr. walsbyi* DSM 16790 grown in complex medium further substantiated PHA accumulation by this strain, which was confirmed by ^1^H-NMR studies of fresh cells, evidencing the accumulation of PHB homopolyester, which, however, did not exceed 0.1% of CDM [69]. In 2011, the strain was grown aerobically with illumination on a medium containing 195 g/L NaCl and 0.5 g glycerol, 0.1 g yeast extract and 1 g sodium pyruvate as carbon sources; atomic force microscopy (AFM) was used for a detailed study of the cellular morphology. Importantly, these AFM studies showed corrugation of the cellular surface due to the presence of PHA granules, which were of almost uniform size within a single cell, and were packaged in tight bags. It was assumed that the primary function of these PHA granules was to reduce the cytosol volume, thus reducing the cellular energy demand for osmotic homeostasis; hence, they play a pivotal role for the strain to cope with the high salinity. In the supplementary material, the authors provided also impressing fluorescence microscope pictures of the cells with PHA granules visible as stained inclusions [88].

### 7.7. Halococcus *sp.*

A total of 20 haloarchaeal strains from strain collections were screened by Legat et al. via different PHA-staining techniques (Sudan Black B, Nile Blue A, and Nile Red). Both complex and defined cultivation media were used for the experiments. Further, PHA granules were visualized via TEM, while ^1^H-NMR spectroscopy was applied to determine PHA composition. Beside strains known before as PHA producers like *Har. hispanica* DSM 4426^T^ or *Hgr. walsbyi* DSM 16790, other organism like *Hbt. noricense* DSM 9758^T^, *Halococcus (Hcc.) dombrowskii* DSM 14522^T^, *Hcc. hamelinensis* JCM 12892^T^, *Hcc. morrhuae* DSM 1307^T^, *Hcc. qingdaonensis* JCM 13587^T^, *Hcc. saccharolyticus* DSM 5350^T^, *Hcc. salifodinae* DSM 8989^T^, *Hfx. volcanii* DSM 3757^T^, *Halorubrum (Hrr.) chaoviator* DSM 19316^T^, *Hrr. coriense* DSM 10284^T^, *Natronococcus (Ncc.) occultus* DSM 3396^T^, and *Natronobacterium (Nbt.) gregoryi* NCMB 2189^T^ showed for the very first time accumulation of PHA when cultured in defined media with 200 g/L NaCl. By these tests, *Halococcus (Hcc.)* was identified as a new genus of PHA-producing microbes. While *Hcc. saccharolyticus* produced PHB homopolyester, all other strains produced PHBHV copolyesters without a supply of 3HV precursors. In this study, TEM pictures were produced for *Hcc. morrhue* and *Hcc. salifodine*, which showed the presence of at least one PHA carbonosome per cell, each about 0.05 to 0.3 µm in diameter [68].

### 7.8. Halogranum *sp.*

The haloarchaeon *Halogranum (Hgn.) amylolyticum* TNN58 was isolated in 2015 by Zhao and colleagues from marine solar salterns near Lianyungang in PR China. This organism was described to be a proficient producer of PHBHV copolyesters from simple, structurally unrelated substrates without being supplied with 3HV-related precursor compounds. Observed by TEM, a high number of PHA granules were visible inside the cells. High 3HV fractions in PHBHV exceeding 0.2 mol/mol are the up to now highest 3HV content in PHBHV reported for PHBHV copolyester production by wild-type organisms from unrelated carbon sources. Nitrogen limitation turned out to support PHBHV production by the strain *Hgn. amylolyticum* TNN58, though PHBHV accumulation occurred in an at least partially growth-associated way. Among the substrates acetate, benzoic acid, butyric acid, casamino acids, glucose, glycerol, lauric acid, and starch, the use of glucose allowed best biomass growth and highest PHA productivity. Fed-batch cultivations under controlled conditions in 7.5 L bioreactors were performed to investigate PHBHV production by *Hgn. amylolyticum* in more details. After 188 h of cultivation, CDM, PHBHV concentration, PHBHV fraction in biomass, and volumetric PHBHV productivity amounted to 29 g/L, 14 g/L, 48 wt.%, and 0.074 g/(L·h), respectively [89].

### 7.9. Haloterrigena *sp.*

The haloarchaeon *Haloterrigena (Htg.) hispanica* DSM 18328^T^ was originally isolated as strain “FP1”, and was the dominant organism thriving in a saltern crystallizer pond at Fuente dePiedra in the south of Spain. Romano and colleagues were the first who described this strain. The strain needs a minimum salinity of 150 g/L NaCl to grow optimally; growth occurs in a salinity range of 130–230 g/L NaCl, at pH-values between 6.5 and 8.5, and at temperatures between 37 °C and 60 °C. These authors also mentioned accumulation of “PHB” in this organism under nutritionally optimal cultivation conditions; corresponding to their publication, this postulation was made merely based on observation of PHA inclusions in the phase contrast microscope without characterizing the composition of the material at the level of monomers [90]. Later, *Htg. hispanica* DSM 18328^T^ was cultivated by Di Donato and colleagues in a highly saline medium containing 200 g/L NaCl using carrot- or tomato waste, which accrues at enormous quantities in many countries like Italy, as sole carbon sources. This study confirmed that this thermophilic strain grows optimally at 50 °C; using this temperature, the organism was cultivated in batch bioreactor fermentation setups, which lasted five days, and also in dialysis fermentations, where bioreactors were equipped with a dialysis tube. Using a complex cultivation medium, the PHB homopolyester was produced at a quantity of 0.135 wt.% PHB in biomass; product composition was determined by ^1^H-NMR analysis. When using carrot waste as substrate, 0.125 wt.% PHB in biomass were accumulated by *Htg. hispanica*, which is a quantity comparable to results obtained by cultivations on expensive media based on casamino acids and yeast extract. Astonishingly, ^1^H-NMR analysis of this biopolyesters produced from carrot waste medium disclosed that the homopolyester poly(4-hydroxybutyrate), a highly flexible material with broad use in the surgical field, was produced instead of expected PHB [91].

### 7.10. Halorhabdus *sp.*

In 2000, the aerobic organism AX-2T was isolated by Wainø and colleagues from sediments of the Great Salt Lake in Utah, USA. This thermophilic strain grew optimally at extremely high NaCl concentrations of 270 g/L, which that time constituted the highest salinity optimum at all reported for any living species. Further, 50 °C and a neutral pH-value were determined as optimum growth parameters. Only a limited number of carbohydrates, namely glucose, fructose, and xylose, were accepted by the strain for biomass formation, while neither fatty acids nor complex substrates like peptone or yeast extract enabled microbial growth of this strain. Cells of this isolate lyse instantly when exposed to distilled water, and were tested positively for PHA biosynthesis (“PHB is produced”); however, neither quantitative data for PHA production nor PHA composition were reported. Based on the outcomes of 16S rRNA analysis, the strain was classified as member of the Halobacteriaceae, but showed only limited similarity to other described species of this family. The new taxon name *Halorhabdus (Hrd.) utahensis* was selected for this new strain, which is now deposited as DSM 12940^T^ [92]. *Hrd. tiamatea* is another representative of this genus. This extremely halophilic, non-pigmented archaeon was isolated in 2008 by Antunes and colleagues from a hypersaline, anoxic deep-sea brine-sediment interface of the Northern Red Sea, an unusual athalassohaline environment associated with tectonic activity. Also *Hrd. tiamatea* revealed optimal growth at a salinity of 270 g/L NaCl, neutral pH-value, a temperature of 45 °C, and the conversion of starch for biomass formation. In contrast to *Hrd. utahensis*, which can be cultivated under both aerobic and anaerobic conditions, *Hrd. tiamatea* shows a clear preference for microaerophilic environments. However, the fact that *Hrd. tiamatea* accumulates PHA was revealed merely as a short annotation in this publication (“Poly-β-hydroxybutyrate is produced”); for this PHA-production test, based only on observation in phase-contrast microscope, cells were cultivated in HBM minimal medium supplemented with 0.005% (*w*/*v*) NH_4_Cl and 0.5 to 1% (*w*/*v*) maltose [93]. Later, the same group of authors deciphered the complete genome of *Hrd. tiamatea*, which disclosed significant differences to the genome of *Hrd. utahensis*; for example, it was revealed that *Hrd. tiamatea* possesses putative trehalose and lactate dehydrogenase synthase genes, which are not found in *Hrd. utahensis* [94]. Finally, the facultative anaerobic strain *Hrd. rudnickae*, isolated from a borehole sample taken at a Polish salt mine, is the third member of the genus *Halorhabdus*, which was described to accumulate PHA. This organism forms non-motile Gram-negative cocci, is red pigmented, und thrives best at a salinity of 200 g/L NaCl, a temperature of 40 °C and a neutral pH-range. Again, PHA inclusions in cells were spotted by TEM, but neither quantified nor characterized (“Poly-β-hydroxybutyrate is produced”) [95]. Hence, production of PHA biopolyesters by the *Halorhabdus* genus is still awaiting its kinetic analysis and characterization at the monomeric level.

## 8. Conclusions

As detailed in the present review, a two-digit number of different haloarchaeal species were already described as potential PHA producers. However, most of these studies were restricted to modest cultivation scales, often merely reporting on microscopic observation and fluorescence staining of PHA granules. To the best of the author´s knowledge gained from the open literature and discussions with other scientists active in this field, there are not more than four haloarchaeal species (*Hfx. mediterranei*, *Hpg. aswanesnsis*, *Hgn. amylolyticum*, and *Htg. hispanica*), for which PHA accumulation was studied in cultivations performed under controlled conditions in bioreactors. However, such bioreactor cultivation setups are the conditio sine qua non to get reliable kinetic data, and reasonable amounts of product for in-depth characterization. Most of all, sufficient amounts of product are needed for processing it to marketable prototype specimens; such processing is completely lacking in the case of haloarchael PHA. Moreover, techno-economic assessment of PHA production by haloarchaea, based on solid experimental data and holistic consideration of the entire production cycle, is only available for *Hfx. mediterranei*, for which economic and life cycle considerations were carried out based on the surplus substrates whey and waste stillage. Nevertheless, exactly these early techno-economic assessments already indicate the high potential of the extremely halophilic members of the Archaea domain for bio-economic biopolyester production of the future. Taking advantage of the broad substrate spectrum, the formation of PHA heteropolyesters of tunable composition and microstructure in dependence on the cultivation strategy, the accessibility of haloarchaea towards inexpensive and convenient product recovery from biomass, the recyclability of process side-streams (spent fermentation broth and cell debris), the detailed knowledge about the complete genome of an increasing number of haloarchaea, and the expedient robustness of such cultivation batches sets haloarchaea at the forefront of efforts dedicated to finally make PHA economically competitive polymers with plastic-like properties, which also match the end-consumer´s expectations. What is needed now is upscaling those processes at a promising lab-scale, and to tap the wealth of haloarchaea reported to produce PHA merely on a qualitative basis, or which have not yet been studied for PHA biosynthesis. In addition, one should be aware of parallel R&D activities with halophilic eubacteria as PHA production strains; here, especially the seminal works with *Halomonas bluephagenensis* TD01 should be mentioned, a proficient PHA production strain which can be cultivated in open bioreactor facilities [96], and which is well studied in terms of genetic manipulation [97,98]. Other examples for promising halophilic eubacteria as PHA producers encompass *Halomonas halophila* [99], or *Halomonas campaniensis* [100]. However, these organisms thrive best under salinities of about 60–70 g/L, which is drastically below the optimum salinity of haloarchaea, which makes the long-term stability of fermentation batches with *Halomonas* sp. uncertain compared with their “competitors” from the realm of haloarchaea.

To summarize the aforementioned, Table 1 and Table 2 provide an overview of the PHA production processes by the individual haloarchaea discussed in the review, indicating the productivities, type of biopolyester produced, and studied production scale. While Table 1 collects the setups on smaller scale, Table 2 refers to the rather scarce number of setups carried out under controlled conditions in laboratory and pilot scale bioreactors.

## Figures and Tables

**Table 1:** PHA production by haloarchaea on shaking flask and stirred flask scale—collected data from literature.

Species	Strain Isolation	Salinity in Medium, Substrates, T	Product	Production Scale/Productivity	Ref.
***Hfx. mediterranei***	Salt pond at the coast near Alicante, Spain	150 g/L NaCl Molasses wastewater T = 15, 20, 25, and 35 °C	PHBHV (16.7 mol.% 3HV)	2.5 L aerated and stirred flasks 0.62 (g/L·h), q_Pmax._ = 0.037 1/h (35 °C)	[46]
***´´***	´´	200 g/L NaCl; T = 37 °C 25–50% pre-treated vinasse	PHBHV (12.4 mol.% 3HV using 25% vinasse) (14.1 mol.% 3HV using 50% vinasse)	Shaking flask scale; 19.7 g/L PHA, 70 wt.% PHA in CDM, 0.21 g/(L·h)	[54]
***´´***	´´	200 g/L NaCl; T = 37 °C Rice-based stillage	PHBHV (15.3 mol.% 3HV)	Shaking flask scale; 16.4 g/L PHA, 70 wt.% PHA in CDM, 0.17 g/(L·h)	[51]
***´´***	´´	190 g/L total salts; 144 g/L NaCl; Alkaline hydrolyzed *Ulva* sp. (macroalgae) as substrate T = 42 °C	PHBHV (3 mol.% 3HV)	Shaking flask scale; batch cultivation; 2.2 g/L, 58% PHA in CDM, 0.035 g/(L·h)	[55]
***´´***	´´	220 g/L NaCl Dephenolized and native olive mill waste water (OMW) T = 37 °C	PHBHV (6.5 mol.% 3HV)	Shaking flask scale, batch cultivation; 43 wt.% PHA in CDM (concentration and productivity data inconsistent in publication)	[53]
***´´***	´´	156 g/L NaCl; Glucose; nitrate or ammonia as N-source T = 37 °C	PHBHV (12.5 mol.% 3HV using nitrate) (16.9 mol.% 3HV using ammonia)	Shaking flask scale, batch cultivation; 0.63 g/L, 4.6% PHA in CDM, 0.035 g/(L·h) with ammonia (C/N = 8) 0.80 g/L, 9.3% PHA in CDM, 0.035 g/(L·h) with ammonia (C/N =8)	[44]
***´´***	´´	156 g/L NaCl; T = 37 °C Glucose; varying phosphate concentrations	PHBHV (22.4 mol.% 3HV)	500 mL shaking flasks, batch 0.95 g/L PHA, 15.6 % PHA in CDM; 0.007 g/(L·h) with optimum phosphate concentration 0.5 g/L KH_2_PO_4_	[45]
***´´***	´´	156 g/L NaCl; Different even- or add-numbered fatty acids T = 37 °C	PHBHV (random or *b*-PHBHV) (˂10 mol.% 3HV using even-numbered acids) (>87 mol.% 3HV using odd-numbered acids)	Shaking flask scale; batch and fed-batch 0.4–1.5 g/L PHA, 10.3–27.1 wt.% PHA in CDM, 0.003–0.010 g/(L·h) (fed-batch, dependent on C-source)	[66]
***Hfx. volcanii***	Dead Sea	200 g/L NaCl; T = 37 °C Glucose	?	Shaking flask scale; Below detection limit	[68]
***´´***	´´	250 g/L NaCl; T = 37 °C Glucose + yeast extract	“PHB”	Shaking flask scale; 7 wt.% PHA in CDM	[29]
***´´*** (strain BBK2)	Solar salterns of Ribandar in Goa, India	200 g/L NaCl; T = 37 °C Sugarcane bagasse hydrolysate	? (not identified)	Shaking flask scale; Not quantified	[78]
***Hfx. gibbonsii***	Salt pond at the coast near Alicante, Spain		“PHB”	Shaking flask scale; 1.2 wt.% PHA in CDM	[29]
***Har. marismortui***	Dead Sea	200 g/L NaCl; Raw and charcoal-pretreated vinasse from bioethanol production	PHB	Shaking flak scale; PHA content in CDM between 23 wt.% (10% non-detoxified vinasse) and 30 wt.% (100% charcoal-detoxified vinasse); 0.015 (non-detoxified) and 0.02 (detoxified) g/(L·h) PHB (2.8 and 4.5 g/L PHB, respectively)	[74]
***Har. hispanica***	Salt pond at the coast near Alicante, Spain	250 g/L NaCl; T = 37 °C Glucose + yeast extract	“PHB”	Shaking flask scale; PHA content: 2.6 wt.% PHA in CDM	[29]
***´´***	´´	200 g/L NaCl; T = 37 °C Glucose	PHBHV	0.09 wt.% PHA in CDM	[68]
***Har.*** **sp. IRU1**	Hypersaline Urmia lake, Iran	250 g/L NaCl 42 °C (other T tested) Glucose (other substrates tested)	PHB	Shaking flask scale; 66 wt.% PHB in CDM	[69]
***´´***	´´	250 g/L NaCl 42 °C (other T tested) Glucose (other substrates tested)	PHB	Shaking flask scale; 62 (glucose), 57 (starch), 56 (sucrose), 55 (fructose), 40 (acetate), 39 (palmitic acid) wt.% PHB in CDM Max. PHA concentration and productivity: 0.98 g/L, 0.016 g/(L·h) (glucose)	[70]
		250 g/L NaCl 47 °C (other T tested) Petrochemical wastewater, tryptone	PHB	Shaking flask scale; Max. 46.6 wt.% PHB in CDM (2% petrochemical wastewater, yeast extract, 47 °C	[71]
***´´***	´´	250 g/L NaCl 47 °C (other T tested) Crude oil, yeast extract (other N-sources tested)	PHB	Shaking flask scale; Max. 41.3 wt.% PHB in CDM (2% crude oil, yeast extract, 47 °C)	[72]
***Har. Japonica*** (strain BS2)	Solar salterns of Ribandar in Goa, India	200 g/L NaCl; T = 37 °C Sugarcane bagasse hydrolysate	? (not identified)	Shaking flask scale; Not quantified	[78]
***Hgm. borinquense*** (strain TN9)	Solar salterns of Marakkanam in Tamil Nadu, India	200 g/L NaCl; T = 37 °C Glucose	PHB	Shaking flask scale; PHA content in CDM 14 wt.%; ca. 3 mg/(L·h) PHA	[76]
***Hgm. Borinquense*** (strain E3)	Solar salterns of Marakkanam in Tamil Nadu, India	200 g/L NaCl; T = 37 °C Glucose	PHBHV (21.5 mol.% 3HV)	Shaking flask scale; PHA content in CDM 74 wt.%; 0.21 g/(L·h) PHA	[77]
***´´***	´´	200 g/L NaCl; T = 37 °C 25% and 50% hydrolyzed sugarcane bagasse	PHBHV (13.3 mol.% 3HV)	Shaking flak scale; PHA content in CDM between 45 and 50 wt.%; 0.0113 g/(L·h) PHBHV on 25%	[78]
***´´***	´´	200 g/L NaCl; T = 37 °C Starch and carbon-rich fibrous waste (cassava bagasse)	PHBHV (13.1% 3HV with starch, 19.7% 3HV with cassava waste)	Shaking flask cultivations in batch mode; Starch: 4.6 g/L PHA, 0.02 g/(L·h), 74.2% PHA in CDM, Cassava bagasse: 1.52 g/L, 0.006 g/(L·h), 44.7% PHA in CDM	[79]
***Hbt. noricense***	Bore core of an Austrian Permian salt deposit	200 g/L NaCl; T = 37 °C Glucose	PHBHV	Shaking flask scale; 0.11 wt.% PHA in CDM	[68]
***Hcc. dombrowskii***	Dry rock salt from Austrian alpine salt mine	Complex saline medium; T = 37 °C	PHBHV	Shaking flask scale; 0.16 wt.% PHA in CDM	[68]
***Hcc. hamelinensis***	Stromatolites from the Hamelin pool in the Australian Shark Bay	Complex saline medium; T = 37 °C	PHBHV	Shaking flask scale; Not quantified	[68]
***Hcc. morrhuae***	Dead Sea	Complex saline medium; T = 37 °C	PHBHV	Shaking flask scale; Not quantified	[68]
***Hcc. qingdaonensis***	Crude sea-salt sample collected near Qingdao, PR China	Complex saline medium; T = 37 °C	PHBHV	Shaking flask scale; Not quantified	[69]
***Hcc. saccharolyticus***	Salt; Cadiz, Spain	Complex saline medium; T = 37 °C	PHB	Shaking flask scale; 1.2 wt.% PHA in CDM	[68]
***Hcc. salifodinae***	Austrian alpine rock salt	Complex saline medium; T = 37 °C	PHBHV	Shaking flask scale; 0.06 wt.% PHA in CDM	[68]
***´´*** (strain BK6)	Solar salterns of Ribandar in Goa, India	200 g/L NaCl; T = 37 °C Sugarcane bagasse hydrolysate	n.d.	Shaking flask scale; Below detection limit	[78]
***Hrr. chaviator***	Sea salt in Baja California, Mexico, Western Australia and Greece	200 g/L NaCl; T = 37 °C Glucose	PHBHV	Shaking flask scale; Not quantified	[68]
***Hrr. coriense***	Dead Sea	200 g/L NaCl; T = 37 °C Glucose	PHBHV	Shaking flask scale; Not quantified	[68]
***Hbf. Haloterrestris*** (“strain 135(T)”)	Samples collected from surface of hypersaline soil collected in Aswan, Egypt	220 g/L NaCl; T = 42 °C (other T tested) Acetate + butyric acid or complex media	PHB	Shaking flask scale; 40 wt.% PHB in CDM on butyric acid, 15 wt.% PHB in CDM on complex medium	[83]
***Nnm. ajinwuensis (=altunense)***	Indian salt production pans	200 g/L NaCl (other salinities tested); T = 37 °C Glucose	PHBHV (13.9 mol.% 3HV)	Repeated batch cultivations in shaking flaks PHA content in CDM 61 wt.%; ca. 15 g/L PHA; 0.21 g/(L·h) PHA	[61]
***Nnm. Palladium*** (strain JCM 8980, =isolate 1KYS1)	Kayacik saltern, Turkey	250 g/L NaCl; Starch	PHBHV (25 mol.% 3HV)	Shaking flak cultivations; PHA content in CDM 53 wt.%; 0.3 mg/(L·h) PHA	[84]
***Nbt. gregoryi***	Soda slat lake liquors from the East African Magadi soda lake	200 g/L NaCl; T = 37 °C alkaliphile; Carbohydrates	PHB	Shaking flask scale; 0.62 wt.% PHB	[68]
***Ncc. occultus***	Magadi Lake, Kenia	200 g/L NaCl; T = 37 °C alkaliphile; Glucose	PHBHV	Shaking flask scale; 3.1 wt.% PHB	[68]
***Hrd. utahensis***	Sediments of the Great Salt Lake in Utah	270 g/L NaCl (maximum described salinity optimum for living beings!); T = 50 °C Limited number of carbohydrates	Not specified (“PHB is produced”)	Shaking flask scale; No quantitative data	[93]
***Hrd. tiamatea***	Hypersaline, anoxic deep-sea brine-sediment interface of the Red Sea	270 g/L NaCl (maximum described salinity optimum for living beings!); T = 45 °C Starch	Not specified (“PHB is produced”)	Shaking flask scale; No quantitative data	[94]
***Hrd. rudnickae***	Borehole at Polish salt mine	200 g/L NaCl; T = 40 °C	Not specified (“PHB is produced”)	Shaking flask scale; No quantitative data	[96]
***Hqr. walsbyi***	Sinai peninsula and saltern crystallizers in Australia and Spain	140–360 g/L NaCl for growth (optimum: >180 g/L); T = 25–45 °C	PHB	Shaking flaks scale; ˂1 wt.% PHA in CDM	[68,88,89]

**Table 2:** PHA production by haloarchaea on bioreactor scale—collected data from literature.

Species	Strain Isolation	Salinity in Medium, Substrates, T	Product	Production Scale/Productivity	Ref.
***Hfx. mediterranei***	Salt pond at the coast near Alicante, Spain	250 g/L marine salts Starch (20 g/L) Glucose (10 g/L) T = 38 °C (other T tested)	PHBHV (in publication: “PHB”)	Stable (monoseptic) continuous cultivation over 3 months in 1.5 L bioreactor; 6.5 g/L PHA on starch 3.5 g/L on glucose	[32]
*´´*	´´	150 g/L NaCl; T = 37 °C Glucose plus yeast extract	PHBHV (10 mol.% 3HV)	10 L bioreactor; fed-batch feeding; 0.21 g/(L·h), 13 g/L PHA, 0.7 g PHA in CDM	[37]
*´´*	´´	200 g/L NaCl; T = 37 °C Hydrolyzed whey permeate Hydrolyzed whey permeate plus GBL	PHBHV (6 mol.% 3HV) P(3HB-*co*-3HV-*co*-4HB) (21.8 mol.% 3HV, 5.1 mol.% 4HB)	42 L bioreactor fed-batch process; 0.09 g/(L·h), 12.2 g/L PHBHV 0.14 g/(L·h), 14.7 g/L poly(3HB-*co*-3HV-*co*-4HB)	[47]
*´´*	´´	150 g/L NaCl; T = 37 °C Hydrolyzed whey permeate	PHBHV (10 mol.% 3HV)	200 L fed-batch **pilot process** (300 L bioreactor); **techno-economic assessment** 7.2 g/L PHA, 66 wt.% PHA in CDM, 0.11 g/(L·h)	[58,59]
*´´*	´´	200 g/L NaCl; T = 37 °C Hydrolyzed whey permeate, spent fermentation broth and saline cell debris from previous whey-based processes	PHBHV (10 mol.% 3HV)	10 L bioreactor batch process 0.04 g/(L·h), 2.28 g/L PHA	[58]
*´´*	´´	156 g/L NaCl; T = 37 °C Hydrolyzed whey permeate, elevated trace element concentration	PHBHV (˂2 mol.% 3HV)	2 L bioreactor batch process 8 g/L PHBHV, 0.17 g/(L·h), 53 wt.% PHA in CDM	[48]
*´´*	´´	150 g/L NaCl; T = 37 °C CGP; CGP plus GBL	PHBHV (10 mol.% 3HV) P(3HB-*co*-3HV-*co*-4HB) (11 mol.% 3HV, 5 mol.% 4HB)	42 L/10 L bioreactor fed-batch process; 0.12 g/(L·h), 16.2 g/L PHA 0.10 g/(L·h), 11.1 g/L PHA	[33]
*´´*	´´	200–230 g/L NaCl; T = 37 °C Native cornstarch treated via enzymatic reactive extrusion	PHBHV (10.4 mol.% 3HV)	6 L bioreactor pH-stat fed-batch process; 0.28 g/(L·h), 0.508 g PHA in CDM; 20 g/L PHA	[49]
*´´*	´´	234 g/L NaCl; T = 37 °C Mixtures of extruded rice bran plus extruded cornstarch	PHBHV (about 11 mol.% 3HV)	5 L bioreactor; pH-stat feeding strategy; 77.8 g/L PHA	[50]
	´´	200 g/L NaCl; Rice-based stillage T: n.r,	PHBHV (17.9 mol.% 3HV)	Unsterile 50 L plug-flow PMMA bioreactor; **techno-economic assessment** 13 g/L PHA, 63 wt.% PHA in CDM, 0.14 g/(L·h)	[52]
*´´*	´´	156 g/L NaCl; Mixes of butyric & valeric acid; Tween80 T = 37 °C	PHBHV (43 mol.% 3HV at butyric/valeric acid = 56/44)	Fed-batch bioreactor cultivation 4.01 g/L PHA, 59 wt.% PHA in CDM; 0.01 g/(L·h)	[65]
*´´* (EPS-negative mutant; strain “ES1”)	´´	140 g/L total salts (110 g/L NaCl) Glucose and valerate T = 37 °C	*b*-PHBHV (up to 50 mol.% 3HV at end of fermentation)	7 L fed-batch bioreactor cultivation Results only reported for shaking flask experiments: max. ca. 5 g/L PHA, 50 wt.% PHA in CDM; 0.17 g/(L·h)	[64]
***Hgr. amylolyticum***	Tainan marine solar saltern near Lianyungang, PR China	200 g/L NaCl; T = 37 °C Glucose	PHBHV (>20 mol.% 3HV)	7.5 L bioreactor; fed-batch feeding strategy; 0.074 g/(L·h), 14 g/L PHBHV, 48 wt.% PHA in CDM	[89]
***Hpg. Aswanensis*** (“strain 56”)	Samples collected from surface of hypersaline soil collected in Aswan, Egypt	250 g/L NaCl; T = 40 °C Sodium acetate and butyric acid	PHB	Corrosion-resistant 8 L composite bioreactor; batch feeding; 0.0045 g/(L·h), 53 wt.% PHB in CDM, 4.6 g/L PHB, 0.018 g(/L·h)	[80]
***Htg. hispanica***	Saltern crystallizer pond at Fuente de Piedra saline lake, Malaga, Spain	200 g/L NaCl; T = 37 °C Complex medium Carrot waste	PHB (complex medium) P(3HB-*co*-3HV-*co*-4HB) (carrot waste)	Bioreactor; batch setups and bioreactor equipped with ultrafiltration unit 0.135 wt.% PHA in CDM (complex medium); 0.125 wt.% PHA in CDM (carrot waste)	[91]
